# OTME-10. Integrated analysis of human gliomas at the single cell level identifies S100A4 as a novel immunotherapy target

**DOI:** 10.1093/noajnl/vdab070.061

**Published:** 2021-07-05

**Authors:** Nourhan Abdelfattah, Parveen Kumar, Jia-Shiun Leu, William Flynn, David Baskin, Kumar Ptichumani, Omkar Ijare, Joshy George, Kyuson Yun

**Affiliations:** 1 Houston Methodist Research Institute, Houston, TX, USA; 2 The Jackson Laboratory, Farmington, CT, USA; 3 Weill Cornell Medical College, New York, NY, USA

## Abstract

Understanding the immune composition of a given tumor is critical to assess its potential responsiveness to cancer immunotherapy. This is especially true for tumors that are intrinsically resistant to immunotherapies, such as GBM. Unfortunately, studies on the functional heterogeneity and associated molecular targets of immune-suppressive cells in vivo have been lacking. Here we report an integrated multi-dimensional analysis of the mutational profiles and single-cell transcriptomics of 60,024 glioma and stromal cells from 16 human samples. We identified molecular signatures of seven distinct macrophage subtypes, each with prognostic clinical value. The three inflammatory subtypes showed hallmarks of TNF/NFκB pathway enrichment and are associated with good outcomes; in contrast, four immunosuppressive subtypes with metabolic pathway hallmarks (oxidative phosphorylation, PI3K/AKT/mTOR, fatty acid metabolism) are associated with poor survival. In addition, we resolved an ongoing controversy in the field regarding the roles of brain resident macrophages, microglia, vs. bone marrow derived macrophages (BMDM) in gliomas. Our data show compelling evidence that microglia are pro-inflammatory and are associated with good survival while BMDMs are mostly immune-suppressive and associated with poor survival. In addition, deciphering immune-suppressive macrophage and Treg molecular signatures enabled us to identify previously unknown immunotherapy targets. In a proof of principle study, we showed that S100A4, a calcium binding protein previously shown to mediate metastasis, was universally upregulated in both innate and adaptive immune suppressor cells, and implantation of gliomas in S100a4-/- host mice significantly extended survival and resulted in pro-inflammatory immune landscape, compared to same glioma cells implanted in B6 control hosts. This functional validation study shows that S100A4 is a highly promising therapeutic target for GBM immunotherapy.

